# Relations Between Executive Functions and Reading Comprehension: A Study of Fourth-Grade Students with and Without Reading Comprehension Difficulties

**DOI:** 10.3390/brainsci14121174

**Published:** 2024-11-23

**Authors:** José-Pablo Escobar, Victoria Espinoza, Sofia Balboa

**Affiliations:** Centro de Desarrollo de Tecnologías de Inclusión, Escuela de Psicología, Pontificia Universidad Católica de Chile, Santiago 8320165, Chile; victoria.espinoza@cedeti.cl (V.E.); sofiadbalboaz@gmail.com (S.B.)

**Keywords:** executive functions, reading comprehension, reading difficulties, working memory, inhibition, cognitive flexibility

## Abstract

Background: The role of non-linguistic factors, such as executive functions, in the reading comprehension process has been analyzed. The present research sought to investigate the relationship between executive functions and reading comprehension. Methods: In an exploratory cross-sectional study, a group of 89 fourth-grade students were evaluated, considering a balanced number of children with and without reading comprehension difficulties. Results: The results indicate that students with reading comprehension difficulties present a lowered profile with respect to both executive functions and reading variables. The path analysis indicates the presence of differences in the variables that explain reading comprehension for both groups. While in the case of students without reading comprehension difficulties, working memory has both a direct and an indirect effect, i.e., through vocabulary, on reading comprehension; in the case of children with reading comprehension difficulties, only cognitive flexibility has a direct impact. In both cases, inhibition has an indirect impact through vocabulary. Conclusions: We reflect on the differentiated role of executive functions according to the level of development of reading skills, highlighting the possibility that some skills may act in a compensatory manner in the presence of general difficulties. The role of vocabulary in the relationship between executive skills and reading is highlighted.

## 1. Introduction

Deficits in reading comprehension not only have an immediate effect on school performance [1] but also on social, occupational, and daily life activities [2]. Therefore, it is necessary to deepen our understanding of the cognitive processes involved in reading comprehension (hereafter, RC) in order to better address its difficulties. In this regard, the psychology of reading has made important advances with respect to understanding the cognitive processes underlying the performance of reading tasks. For example, research shows the importance of decoding for RC in the early stages of reading [3,4]. As mastery of the grapheme–phoneme relationship is achieved, decoding becomes more automatic, bringing fluency to the process and freeing up cognitive resources in favor of reading comprehension [5,6,7]. However, there is a group of students who, despite having adequate decoding and fluency skills, are not able to comprehend what they read. This group has specific reading comprehension difficulties and represents 3% to 10% of the school population [8,9]. This type of profile shows that fluency and decoding are necessary but not sufficient elements for RC, and although linguistic variables play a relevant role in explaining RC [8,9], they are not the only ones; there may be other variables, such as executive functions, that could contribute to the explanation of RC difficulties [10,11].

Executive functions (hereafter, EFs) are defined as a set of cognitive abilities that allow for the planning, executing, and monitoring of goal-oriented tasks [12]. They allow for inhibiting distractors or behaviors unrelated to task execution, maintaining and manipulating information for short periods of time, as well as actively flexing between different task elements or goal-oriented strategies [13]. Evidence has demonstrated the role of EFs in school learning [14,15,16], and the direct and indirect effect of EFs on RC have been especially explored [17,18]. Evidence also shows that EFs are important predictors of later reading comprehension achievement, even after controlling for the effect of word reading, vocabulary, and previous reading comprehension skills [19]. However, the results are inconclusive and scarce for students with reading comprehension difficulties. The purpose of this research is to investigate the effect of the components of executive function (inhibition, working memory, and cognitive flexibility) in their relationship with RC and to evaluate whether these components have different effects on students with and without reading comprehension difficulties

### 1.1. Difficulties in Reading Comprehension

Difficulties in reading comprehension can have different origins. Models such as the simple view of reading [20,21] posit that RC is the product of language skills and decoding. For example, if language skills are adequate but decoding is poor, dyslexia results, where language and decoding skills compromise results in a poor reader profile [20]. In addition, there is the case of poor language skills but good decoding skills, which results in a profile originally called hyperlexia [20], but which has evolved into the concept of poor reading comprehension [22]. What is striking about this profile is that in addition to good decoding there is also adequate fluency, but reading comprehension is nonetheless impaired [23,24,25]. While estimates of the prevalence of this group vary depending on the identification criteria and comprehension test format [9,26], it has been found to be present in 3–10% of the school population [27].

A characteristic of this profile is the adequate development of phonological skills that positively impact decoding [8]; although, in the early stages of reading development, children may experience phonological difficulties, these are overcome early on [28]. Therefore, such difficulties may go unnoticed as they are not evident, especially in school contexts where only the speed component of fluency is assessed over RC, which has implications in terms of identification and treatment [9,24]. While characteristic of this profile of difficulties is the discrepancy between RC skills and decoding [24], other difficulties are also present. This is because RC is a task that requires diverse cognitive processes and skills. For example, researchers have comprehensively analyzed the components of listening comprehension [29] and have reported on deficits in skills such as generating inferences [23,30,31], morphological and syntactic knowledge [32,33,34], and vocabulary [2,35,36]. However, a meta-analysis shows that although language deficits are relevant, they are not necessarily the only origin of these difficulties; they are additive factors [2], offering the possibility of including other explanatory factors in this profile of difficulties such as EFs. This coincides with the proposal of Duke and Cartwright (2021) [37], who suggest the need to expand the SVR model by considering a series of additional factors that may affect RC, including EFs.

As previously described, as one moves through the grades, basic reading skills, such as phonological awareness and decoding, have to yield in their explanatory value for RC performance to higher-order skills such as vocabulary and reading fluency [38]. Evidence is substantial regarding the effect of the automaticity of decoding skills on fluency and subsequent reading comprehension [39,40], as well as the role that EFs may play in this relationship [41,42]. However, fluency is a necessary but not sufficient component for comprehension, as established in the profile of students with difficulties in RC. Therefore, it is necessary, in explaining these difficulties, to explore the role of EFs in explaining difficulties in RC as an additive component.

### 1.2. Executive Functions and Reading Comprehension

Executive functions are defined as a set of high-level cognitive processes that allow for the controlling of other lower-level processes in order to regulate goal-oriented behaviors and thoughts [43]. Although, in the field of the study of EF, there are several explanatory models and new perspectives [44,45], the tripartite model, hierarchically composed of inhibition, working memory, and cognitive flexibility [13], is widely accepted and serves as the foundation for much of the research in this field. The three EFs proposed by this model are independent but related to each other [43]. This has important implications in terms of the evidence used, as well as the stage in reading development at which this model is explored. The evidence regarding the effect of each of the EFs on students with and without difficulties in RC will be reviewed below.

Inhibitory control includes the ability to inhibit thoughts, actions, or behaviors in the face of the appearance of internal or external stimuli [13,46]. Inhibition is considered as a basal component in the tripartite model of EFs, which implies that the rest of the EFs are developed based on inhibitory control [47]. Inhibition would allow for the ignoring of irrelevant information to enable an adequate mental representation of the text being read [48,49,50]. There is a significant association between inhibitory control and reading comprehension performance [51], proposing that inhibition could act as a predictor skill of RC [41]. However, there is also research that reports no effect of inhibition on RC. For example, Johann et al. [52], in a sample of third- and fourth-grade students, do not find that inhibition is associated with RC. Similar results are also reported by Ober et al. [17], who found an association between inhibition and decoding but not with RC. Along the same lines, a study conducted with fourth graders, although it did not find a direct association between inhibition and RC, found an indirect effect through vocabulary [18]. On the other hand, Christopher et al. [53] found that inhibition does not act as a predictor of either reading comprehension or word reading. Therefore, the results of the relationship between inhibition and RC are not conclusive in students without reading comprehension difficulties.

It has been proposed that due to difficulties at the level of inhibitory control, students with RC difficulties present a greater tendency to use irrelevant information [54,55,56]. In this sense, Borella et al. [54] reported that students with RC difficulties exhibit problems with resistance to interference, though not with inhibition of the dominant response or resisting distractors. Carretti et al. [57] concluded that students with RC difficulties present problems in actively maintaining relevant information and inhibiting irrelevant information. Swanson et al. [58] could not identify differences in response inhibition in children with and without reading difficulties. Similar results are reported by Locascio et al. [59], who also found no differences in inhibitory control. Therefore, the study of the effect of inhibition in students with reading comprehension difficulties seems to be inconclusive.

Working memory (WM) is defined as a limited-capacity system that temporarily holds and processes information to support cognitive task performance [60]. WM is the most investigated component of EFs in relation to reading, and especially with respect to RC [53,57,61,62,63]. WM is crucial for reading comprehension because it allows for the retention and manipulation of the information needed to access text meaning and generate inferences [64]. In this regard, Ober et al. [17] reported that WM affects reading comprehension both directly and indirectly through the inference-making ability. This finding highlights inference generation as a mediating variable in the relation between WM and reading comprehension. For fourth-grade students, WM has been reported to have direct effects on reading comprehension, as well as indirect effects through vocabulary, reading fluency, and decoding skills [18]. The results of specific training experiences in working memory have been reported with promising effects on reading comprehension [65]. Likewise, Spencer et al. [66] did not report direct effects of WM on RC but indirect effects through decoding. Direct effects of WM on RC were also not found by Johann et al. [52] in third and fourth graders.

Research that has analyzed the role of WM in students with reading comprehension difficulties has revealed that compared to their peers without difficulties, they show lower performance in WM [27,67]. It has been posited that the deficits of poor comprehenders could be attributed, in part, to difficulties in the WM control mechanisms, which fail especially at the level of verbal processing [57] However, there is also research showing other results. For example, Georgiou and Das [68] found no differences in WM between young adults with and without reading comprehension difficulties. Similar results are also reported by Loscasio et al., [59] who found in a group of students that, despite their having specific deficits in reading comprehension, the students presented a WM at a similar level to controls without difficulties. Therefore, as with inhibition, the evidence for the role of WM in students with reading comprehension difficulties is inconclusive.

Finally, in the hierarchical context of the tripartite model of executive functions, evidence of the role of cognitive flexibility in reading comprehension is presented. Cognitive flexibility is defined as the ability to switch between different subtasks, approaches, or strategies for solving tasks and problems. This ability also allows us to integrate information from different tasks or attentional focus [13]. For example, at a more basic level, cognitive flexibility is necessary to shift attention between the phonological and semantic components needed to comprehend a text [69,70]. In addition, cognitive flexibility has been posited to support RC by allowing readers to consider various aspects of texts simultaneously [71,72].

Multiple research has evidenced the role of cognitive flexibility for reading comprehension [41,73,74]. Kieffer et al. [51] observed that flexibility, as the ability to shift attentional focus, plays multiple roles in RC, both directly and indirectly through linguistic comprehension. In this sense, Spencer et al. [66] report on the indirect effects of flexibility on RC through linguistic skills such as oral comprehension. Escobar and Rosas [41] report that first-grade flexibility is a predictor of third0grade RC, both directly and indirectly through reading fluency. In this sense, Johann et al. [52] also report the direct effect of cognitive flexibility on RC. However, there are also other studies that do not report direct effects of flexibility on RC but indirect effects through vocabulary, which is relevant as it makes the latter a mediator [18].

It has been observed that students with RC difficulties present a decreased performance in cognitive flexibility tasks, even controlling for decoding, verbal ability, reasoning, and vocabulary [75]. However, in a study by Fadaei et al. [76], no significant relation was observed between cognitive flexibility and the presence of reading problems in students with reading difficulties. Although there is considerable evidence regarding the impact of cognitive flexibility on RC, there is also research that has found no effect on this relation. An example of this is Potocki [27], with fifth graders, where no effect of flexibility on RC was found. Similar results are also reported by Ober et al. [17] in young students whose cognitive flexibility skills were neither directly nor indirectly associated through decoding and inference generation with RC. Therefore, the evidence for the relation between cognitive flexibility and RC is not conclusive.

### 1.3. The Current Study

While the literature addresses the effect of executive functions in explaining reading comprehension difficulties, the evidence is still sparse and inconclusive. Moreover, most of the research has been conducted in the orthographic context of English, a language with opaque orthographic qualities that directly affects decoding [77]. Therefore, it is interesting to explore this effect in distinct orthographies, such as Spanish. In this sense, the orthographic qualities of Spanish imprint relevant characteristics for the identification of this type of reading difficulty. This is because grapheme–phoneme correspondences are almost biunivocal [78]; decoding, in general, is not challenging, so many readers achieve—compared to more opaque orthographies—accuracy and fluency earlier in reading development [79]. In practical terms, this means that the identification of reading difficulties in transparent orthographic systems falls more on fluency than on accuracy [80,81]. This is relevant because adequate fluency could mask difficulties in RC and thereby delay the identification of this profile of difficulties.

Additionally, given that in this type of orthographies, the phonological weight for reading would be lower [81,82], the cognitive load would also be lower; therefore, it is possible that EFs such as inhibition and WM could have less weight in the execution of RC tasks, especially in middle school stages, where decoding and fluency skills have reached a certain stability. Therefore, it is possible that the orthographic qualities of Spanish not only affect reading accuracy and speed but may also influence the relation between cognitive processes such as EFs and RC.

The purpose of this research is to investigate the effect of the components of EF, inhibition, working memory, and cognitive flexibility in their relationship with RC and to evaluate whether these components have different effects in students with and without reading comprehension difficulties. It is relevant to know whether the components of EF have different effects based on individual differences in RC as this would allow for a deeper understanding of the difficulties and better target intervention strategies. The fourth grade of primary school is relevant because there is an important change in relation to the teaching of reading comprehension. At this grade, students are expected to have consolidated decoding and fluency, with reading comprehension being the key tool for school learning. Therefore, attention to the cognitive processes involved in students with reading comprehension difficulties is important.

In summary, this research aims to examine whether readers with reading comprehension difficulties also show low performance in other any component of executive function compared to students without such difficulties. In addition, this study aims to identify which components of executive function are directly associated with reading comprehension in students with and without reading difficulties, and which are indirectly associated through skills such as fluency and vocabulary.

## 2. Methods

This is an exploratory cross-sectional study that seeks to identify whether students with reading comprehension difficulties also present difficulties in any of the components of executive function and to identify how the different components of executive function contribute to explaining the variance in reading comprehension between students with and without reading comprehension difficulties. In accordance with the results of previous research, it is expected that students with reading comprehension difficulties will also present some diminished performance in executive functions, and we also predict the existence of differences in the models that explain their overall reading comprehension performance.

### 2.1. Participants

Participants are part of the first cohort of an ongoing longitudinal study of Chilean fourth-grade students from 12 different schools in the Metropolitan Region of Santiago, Chile. Access to the schools has been by convenience as they are schools that have participated in previous research. All of these schools are public. The inclusion criteria required participants to be native Spanish speakers, enrolled in regular schools, and to have obtained informed consent and assent from their parents to participate in the study. Exclusion criteria included school repetition and the presence of sensory, cognitive, or emotional disturbances that could interfere with the assessment process. These factors were reported by the teachers and confirmed by the evaluators during the assessment. All procedures involved in the study were approved by the ethics committee of the University [blinded for review]. The participants were evaluated in the middle of the second semester of the school year, first in a group session lasting 45 min and then individually in a second session scheduled on a different day and lasting 45 min. All the evaluations were carried out in the participants’ own schools. Tests were administered by a group of 6 evaluators trained in education and psychology, and who were also trained in the proper administration of the tests by the principal investigator.

To select participants with reading comprehension difficulties, as well as their controls, their reading comprehension level was assessed, as well as their fluency at the word reading level. Both tests were administered to a sample of 395 participants (mean age = 9.75; *SD* = 0.51, of which 45% were female), who were part of the main study. The participants who obtained a score on the reading comprehension test of more than −1.0 *SD* and average performance on the reading fluency test formed the specific reading comprehension difficulties group (*N* = 89), who represented 22.5% of the original sample. This group had an age *M* = 9.67; *SD* = 0.54, with 30% being female. The control group was randomly selected from the remaining participants who obtained average scores in both reading comprehension and fluency. This group consisted of 89 participants (age *M* = 9.66; *SD* = 0.48, of which 49.4% were female).

### 2.2. Measures

Reading Comprehension: Reading comprehension was assessed through the ELFE II test [83] in its recent standardized version for Latin America [84]. This is a group-administered and time-controlled test. For this research, the scores of the Text Reading Comprehension subtest were used. In this test, participants have 7 min to silently read texts of progressive length and complexity and subsequently identify the correct answer from four possible alternatives. The test has a maximum score of 26 points and a Cronbach’s alpha of 0.98 for fourth-grade students.

Reading Fluency: Word-level reading fluency was assessed through an individually administered test. This is a Spanish adaptation of the Silent Word Reading Fluency Test [85], translated into Spanish and standardized by López-Escribano et al. [86]. In this test, participants have 3 min to identify and segment word strings. For example, the word string “bluedogfly” is presented, and the participant has to segment it with a pencil into “blue/dog/fly”. One point is assigned for each correctly identified word. The test has a maximum of 230 points and adequate test–retest reliability measures of 0.91.

Vocabulary: Vocabulary skills were assessed through the Spanish adaptation of the English Word Generation Vocabulary Test [87] by Meneses et al. [88]. This is an individually administered test, where participants read short statements with a target word underlined within them. The participant must choose, from four alternatives, the synonym that best fits within the context of the sentence. The test has 15 items and is scored 1 point for each correct answer, with a Cronbach’s alpha of 0.80.

Working memory: The verbal component of working memory was evaluated through the digits test of the WISC-V Chilean version [89]. The test is composed of three subtasks. The first subtask is direct digits, and in it, participants have to repeat digits in the same sequence in which they are heard. The second subtask is called digits in reverse order, and in it, participants have to repeat in reverse a sequence of digits heard. Finally, the third subtask is called sequential digits, in which the participants have to repeat the digits heard in sequence from the smallest digit to the largest. One point is awarded for each correctly answered sequence. The test has a maximum total score of 54 points, and the authors report a Cronbach’s alpha of 0.91.

Inhibition: This was evaluated by the Spanish adaptation of the Hayling test [90], translated into Spanish by Pérez et al. [91]. This test is divided into two parts. In the first part, participants have to complete the sentence with a word that makes sense in its semantics and syntax; for example, for the sentence “bees produce ...”, the answer is expected to be the word “honey”. Part B evaluates the inhibition ability since a sentence has to be completed with a word that does not make sense in semantics but preserves the sense in syntax. For example, before the sentence “the hen lays ...”, and the answer is expected to be “fruits”, “candies”, etc. Each correct answer is worth 1 point, and for each wrong answer, 1 point is deducted. The test has a total of 30 points, with a maximum of 15 points for each part and a Cronbach’s alpha of 0.76.

Flexibility: Cognitive flexibility was assessed through the Spanish adaptation of the cognitive flexibility of graphosemantic reading domain task [92]. This task consists of assessing the student’s ability to maintain two mental representations when they are involved in a switching task. The test consists of arranging 12 cards in a 2 × 2 matrix that, in the horizontal plane, have to be classified in the same category, and in the vertical plane, according to their initial sound. For example, a set of two categories (body parts and fruits) and two initials (m and p) with the words hand, apple, foot, pear, etc., is presented, andthe participants have to sort the cards in the shortest possible time in their place. Each correctly sorted card scores 1 point, with 12 points per set. If a card is incorrectly sorted, 1 point is deducted. The total score of the test is 24 points and has a Cronbach’s alpha of 0.82.

### 2.3. Data Analysis

To identify differences in student performance in reading skills and executive functions, a multivariate analysis of variance (MANOVA) was performed. An analysis of the Pearson correlations between the variables was also performed. These analyses were performed with SPSS version 27 statistical software. To analyze the direct and indirect impact of executive functions on reading comprehension, a path analysis was performed. A model is thought to fit the data well when the χ^2^ value is non-significant, the CFI and TLI indices are above 0.95, and the RMSEA and SRMR values are below 0.05 [93]. This analysis was performed using AMOS statistical software version 27.

## 3. Results

### 3.1. Comparissons Between Groups

Table 1 shows the main descriptive statistics of the study variables differentiated between the control group and the group with reading comprehension difficulties (hereafter, RCD).

After checking compliance with the principles of variance homogeneity and homoscedasticity, a multiple analysis of variance (MANOVA) is carried out in order to identify whether there are differences in performance between the groups. The results show that the scores of the RCD group are consistently lower compared to the control group. With respect to inhibition, it is possible to identify that the performance of the RCD group is lower than the control [*F*(1, 174) = 16.357; *p* = 0.000; partial eta squared = 0.085]. Likewise, in the case of WM, the performance of the RCD group is lower than the control [*F*(1, 176) = 31.855; *p* = 0.000; partial eta squared = 0.153]. The results of the analysis of performance in the cognitive flexibility task also show that there are significant differences between the groups [*F*(1, 176) = 4.365; *p* = 0.038; partial eta squared = 0.024]. When analyzing performance with respect to the remaining variables, it is possible to identify that the performance of the RCD group is also lower in vocabulary compared to the control group [*F*(1, 176) = 59.154; *p* = 0.000; partial eta squared = 0.252], as well as in reading fluency [*F*(1, 176) = 23.473; *p* = 0.000, partial eta squared = 0.118] and, as expected, in reading comprehension, with a strong effect size [*F*(1, 176) = 355.879; *p* = 0.000; partial eta squared = 0.669]. The MANOVA shows that in the RCD group, in addition to the low performance in reading comprehension, the participants obtained lower scores in the measures of EF, vocabulary, and fluency compared, to the control group.

### 3.2. Correlations

After exploring the differences in performance, we proceed to analyze the direct and indirect effect of the components of EF on RC performance depending on whether or not there are difficulties. Table 2 shows the correlations between the variables differentiated by group.

With respect to the control group, all variables correlate significantly and moderately with RC, with WM being the executive component that shares the most variance in performance. On the other hand, in the RCD group, cognitive flexibility is the only component of EF that is moderately associated with RC performance. Again, vocabulary is a variable that is related to RC performance in both groups but shares more explanatory variance of RC in the control group. It should be noted that although the gender variable was tested for in all analyses, this variable was not significant in any of them. Therefore, it is not included in the models.

**Figure 1:**
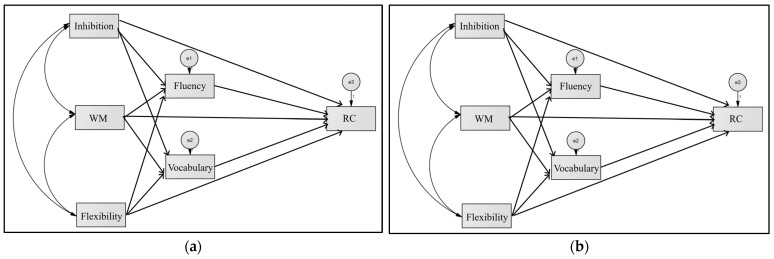
Proposed path analysis models: (**a**) students without reading comprehension difficulties; (**b**) students with RCD.

In order to assess the direct and indirect effects of the components of EF on the RC of students with and without reading difficulties, two path analysis models are proposed. Figure 1 shows the proposed models.

**Figure 2:**
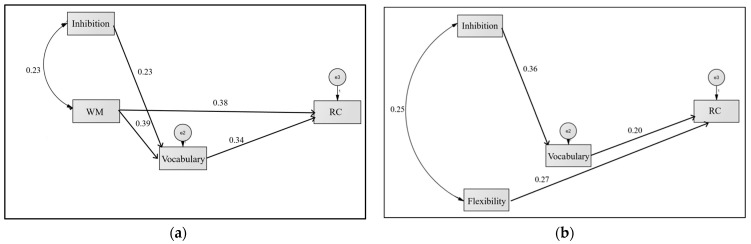
Final model for: (**a**) students without reading comprehension difficulties; (**b**) students with RCD.

The results of the path analysis show that the originally proposed model for the group without RC difficulties does not fit the data. It is therefore necessary to make adjustments to the initial model. Figure 2a shows the final model which does have an adequate fit (χ^2^ = 2.018; *df* = 1; *p* = 0.155; *RMSEA* = 0.10; *CFI* = 0.985; *TLI* = 0.908). This model shows that WM is the executive component directly associated with RC (β = 0.38, *p* < 0.001), as well as indirectly through vocabulary (β = 0.13, *p <* 0.000). Inhibition has only an indirect effect on RC through vocabulary (β = 0.08, *p <* 0.006).

With regard to the model that tries to explain the effect of the components of EF on students with RCD, the original model proposed does not fit the data either. Therefore, it is necessary to make adjustments to the initial model. In the same figure (Figure 2b), it is possible to observe the model that best fits the data (χ^2^ = 3.55; *df* = 2; *p* = 0.169; *RMSEA* = 0.094; *CFI* = 0.950; *TLI* = 0.839). In this model, it is possible to identify that flexibility is directly associated with RC (β = 0.27, *p <* 0.05), while inhibition indirectly through vocabulary (β = 0.07, *p <* 0.05).

## 4. Discussion

This research set out to explore the level of development of executive functions in fourth-grade students with and without reading comprehension difficulties, as well as to evaluate the direct and indirect effect of the components of EF as a function of the presence or absence of difficulties in RC.

In relation to the first objective, the results show that the RCD group presents a lower performance in executive functions compared to the control group. This result confirms what has been observed by other research, where low performance in EFs is also reported in students with RCD [27,55,94]. These results could suggest a causal relationship between low performance in executive components and deficits in RC, although this hypothesis cannot be confirmed in this study due to its cross-sectional nature. In this regard, it has been reported that students with impaired inhibitory control may be less resistant to distractions when performing reading comprehension tasks [54], as well as less able to identify and inhibit irrelevant information when reading [54,55,56]. It has also been reported that low WM performance could compromise the amount of information stored during reading and consequently affect the ability to generate inferences, compromising RC [64]. In turn, research that has explored the role of cognitive flexibility mentions that it could have implications at several levels in reading, from the alternation between phonological and semantic components that allow for RC [69,70] to the use of reading strategies that allow for the consideration of different aspects of the text simultaneously [71,72]. Therefore, poor performance in EFs could be an additional factor in explaining reading comprehension difficulties.

Looking forward to the relation of other factors to RC, we can see that with respect to fluency and vocabulary, the results of this study show that students with RCD also perform poorly compared to students without difficulties on both variables. The evidence for the role of vocabulary in reading comprehension is extensive [95,96], and our results are consistent with the evidence of low performance in students with RCD [2,67]. However, in the path analysis model, fluency was not found to be a mediating variable between EF and RC in either group. Previous research has found that fluency is a mediating variable between inhibition and RC in typical Spanish readers [41]. It is possible that the null effect reported in this research is due to the nature of the reading fluency task used. Unlike Escobar and Rosas [41], who used a fluency indicator based solely on the number of words correctly decoded in one minute, our task considers not only decoding speed but also an important semantic component. In our case, participants must recognize a picture and then read the correct word associated with that picture between alternatives. This is also why fluency and RC were not correlated in the RCD group because the fluency task taps a semantic component. While the evidence regarding the role of fluency for RC is extensive [97], especially as a variable that accounts for automaticity in word recognition that could allow for the freeing of resources in WM in favor of RC [7,97], it is also relevant that there is a dissociation between fluency and RC skills [35,98] and that difficulties in RC may be distinct from difficulties in reading fluency [99].

Regarding the second aim of this study, which is related to the direct and indirect effect of executive components on RC, path analyses show that in the control group, both inhibition and WM are variables associated with RC performance. This finding is relevant and adds to the body of evidence confirming the effect of executive components for reading comprehension [41,48,49,50,51,53,57,61,62]. Specifically, WM was found to be a variable directly associated with RC, which also adds to evidence from others research confirming the fundamental role of WM for RC [53,57,61,62,63].

Surprisingly, it was not possible to find a direct association between WM and RC in students with RCD, despite it being a variable with broad status in explaining this type of difficulty [53,57,61,62,63]. For example, in an investigation carried out by Sesma et al. [100], an association between WM and RC was found in students with RCD. Regarding students with RCD, it has been observed that they present a deficit at the WM level, which coincides with what was observed in the present research [27,67]. In this context, the scarce role observed in the relationship between both variables could be explained by the presence of a very low level of WM, which would determine the preponderant action of mechanisms associated with the rest of the components of EF, especially cognitive flexibility. The importance of this variable could perhaps be related to the integrated nature of the basic components of EF, where cognitive flexibility could contain all the cognitive resources associated with the rest of the functions [47]. However, there is other research that has not observed a direct effect of WM on RC [52].

Also, the results indicate that WM is a variable indirectly associated with RC through vocabulary. This finding is in line with other research that mentions that vocabulary is a mediating variable between WM and the ability to generate inferences [101]. In this sense, the mediating role of vocabulary is relevant since, in addition to being associated with WM, the results indicate that it acts as a mediator of the relationship between inhibitory control and RC. This is a similar result to that reported by Chrysochoou et al. [102], who found that vocabulary acted as a mediator of the association between WM and RC.

With respect to the observed relationship between cognitive flexibility and RC, while other research has found a direct association between these factors [41,51,73,74], our research was unable to replicate this finding in the group of students without difficulties, despite using assessment tasks similar to those reported by previous research. It is possible that this finding is partly since the cognitive flexibility test used has a phonological component that may be more difficult to consolidate for fourth-grade students, as the task is simpler. In this sense, although in correlation analyses, we found a modest association between flexibility and RC, this relationship is lost after introducing flexibility into a model with other more robust variables, especially inhibition (measured with a verbal task more closely associated with vocabulary on measures of cognitive inhibition) and WM, which were found to be related variables. However, in the case of the group of students with RCD, cognitive flexibility was found to have a direct association with RC. This finding is relevant and it is in line with other studies reporting similar results [73,74]. It is important to question how cognitive variables act in the process of reading comprehension; we have observed a strengthening of the role of cognitive flexibility in the case of students who present a decreased development of executive functions.

Finally, regarding the relationship between inhibition and RC, in the present study, only an indirect relationship could be observed through vocabulary, where vocabulary acts as a mediator, both in the RCD group and in the control group. In part, this association can be explained by the type of task used for the assessment of inhibition, as it is a task with important linguistic components in that participants must inhibit the urge to complete correctly presented utterances orally. However, this gives greater relevance to the outcome as it is considered a more linguistic variable.

## 5. Conclusions

In summary, the results show that the components of EF are valid variables associated with RC, both for students with reading comprehension difficulties and for students without difficulties. While in students without reading difficulties, the role of working memory stands out, in the case of students with RCD, there is a stronger association with cognitive flexibility, which could be explained by the presence of a deficit in working memory. It is important to consider the role of vocabulary, both for children with and without reading difficulties, especially with respect to the well-known relationship between vocabulary knowledge and reading comprehension skills [103].

From a theoretical perspective, these results may have important implications as they provide new evidence regarding explanatory models of reading comprehension. In line with Duke and Cartwright [37], it is necessary to consider new explanatory variables, including executive functions. In this new context, the consideration of possible differences in the explanatory role of executive functions in children with and without RCD is crucial as it allows us to generate as many theoretical reflections as didactic perspectives relevant to the real contexts in which students perform. Our results support the idea that students with RCD also present a lowered profile with respect to executive functions and vocabulary and also propose a new distinction in the role of executive functions on reading comprehension, establishing important differences according to the students’ level of reading comprehension development. The observed differences indicate that in the case of students without RCD, there is greater agreement with the results of previous research, which assign a predominant role to WM. However, the main finding of this study would be the role of CF in the group of children with comprehension difficulties, as this generates a new line of research that could serve both to explain the nature of the difficulties presented by the students and to design didactic strategies appropriate to their needs. It is also fundamental to analyze the scarce role that inhibition—a component that is usually very strengthened in school contexts—manifested, and it would be interesting for future research to investigate this further.

Regarding the practical implications of this study, these results may be useful for the design of intervention strategies in students with RCD, especially when considering the differentiated role played by the different components of executive function for this group of students. This does not imply that the use of RC strategies that promote the use of WM is left aside; rather, it focuses on the need to consider cognitive flexibility as a fundamental axis when developing didactic strategies for students with RCD. It is also important to consider poor performance in EFs in students with RCD from a general perspective because, as they are core cognitive processes, they could compromise the effectiveness of an intervention. For example, it could be essential to control the effect of distracting environmental variables to reduce the demands on inhibitory control [54], or to partialize the delivery of information in order to reduce the cognitive load on working memory [104,105], as well as to make explicit the need to focus on different aspects of the text when reading. While explicit training of EF components has an effect, the results are inconclusive [104,105,106,107,108,109]. Caution should also be exercised as there is evidence that the level of EFs development may be of limited value in predicting which students will respond to intensive reading interventions [110]. Therefore, it is important to make explicit that low performance in executive functions may not necessarily explain reading comprehension difficulties on its own but will do so together with other variables such as vocabulary, as has been identified in this research. Thus, a better development of EFs that allows for the better deployment and allocation of attentional resources would support a more efficient processing of information to build an integrated mental representation of the text and thus an adequate understanding of the message it contains.

These results have to be used with caution as they are restricted to a small sample of Chilean students in the fourth year of primary school, which, although they come from different types of schools, show a partial picture of the development of reading skills. One of the limitations of the study is that being a cross-sectional study, it does not allow us to establish causality between variables. This is relevant since the evidence does not allow us to state whether low performance in executive functions is the cause of difficulties in CR. Finally, although one standard is the use of decoding measures to identify reading difficulties, this is not as necessary in transparent orthographic systems such as Spanish, where fluency measures are more decisive of reading performance than accuracy measures [25]. Future research should consider a longitudinal component in order to establish causal relationships and to determine with greater specificity the nature of the relationships between the different components of executive function and CR. It would also be interesting to investigate the differentiated role of executive functions according to performance in other reading skills, such as fluency and decoding. On the other hand, it would also be interesting to continue investigating the role of working memory in reading comprehension in students with diverse reading performance and to investigate, in greater depth, how this relates to the rest of the components of executive function to support reading comprehension processes.

## Figures and Tables

**Table 1:** Descriptive statistics.

Variable	Control (N = 89)						RCD (N = 89)		
	Min	Max	M	DS	Min	Max	M	DS	*p*
RC	9	25	15.97	4.58	1	8	5.62	1.95	0.000
RF	40	151	82.81	20.56	39	129	64.88	16.74	0.000
Inhi	4	15	11.57	2.53	3	15	10.19	2.61	0.000
WM	4.67	11	7.46	1.36	3.33	11.33	6.29	1.37	0.000
Flex	11	25	22.10	3.36	11	24	20.77	3.57	0.007
Voc	3	15	10.04	2.63	1	12	6.90	2.50	0.000

Note: RC, reading comprehension; RF, reading fluency; Inhi, inhibition; WM, working memory; Flex, flexibility; Voc, vocabulary.

**Table 2:** Correlations between study variables.

Control						
		1	2	3	4	5
1	RC					
2	RF	0.414 **				
3	Vocabulary	0.506 **	0.403 **			
4	Inhibition	0.306 **	0.076	0.317 **		
5	WM	0.530 **	0.412 **	0.440 **	0.226 *	
6	Flexibility	0.281 **	0.134	0.154	0.170	0.321 *
RCD						
		1	2	3	4	5
1	RC					
2	RF	0.103				
3	Vocabulary	0.270 *	0.137			
4	Inhibition	0.109	0.176	0.363 **		
5	WM	0.157	0.233 *	0.172	0.103	
6	Flexibility	0.322 **	0.033	0.267 *	0.248 *	0.113

Note: RC, reading comprehension; RF, reading fluency; WM, working memory. Significant correlations are in bold. * *p* < 0.05; ** *p* < 0.01.

## Data Availability

The data used in the present study are available upon direct request by contacting the corresponding author due to the expensive cost of collecting research data and the involvement of participants’ personal information.
